# Non‐pharmacological interventions for challenging behaviours of adults with intellectual disabilities: A meta‐analysis

**DOI:** 10.1111/jir.12736

**Published:** 2020-06-17

**Authors:** E. Bruinsma, B. J. van den Hoofdakker, A. P. Groenman, P. J. Hoekstra, G. M. de Kuijper, M. Klaver, A. A. de Bildt

**Affiliations:** ^1^ University of Groningen, University Medical Centre Groningen, Department of Child and Adolescent Psychiatry Groningen The Netherlands; ^2^ University of Groningen Department of Clinical Psychology and Experimental Psychopathology Groningen The Netherlands; ^3^ Centre for Intellectual Disability and Mental Health Assen The Netherlands

**Keywords:** Challenging behaviour, Intellectual disabilities, Meta‐analysis, Non‐pharmacological interventions

## Abstract

**Background:**

Non‐pharmacological interventions are recommended for the treatment of challenging behaviours in individuals with intellectual disabilities by clinical guidelines. However, evidence for their effectiveness is ambiguous. The aim of the current meta‐analysis is to update the existing evidence, to investigate long‐term outcome, and to examine whether intervention type, delivery mode, and study design were associated with differences in effectiveness.

**Method:**

An electronic search was conducted using the databases Medline, Eric, PsychINFO and Cinahl. Studies with experimental or quasi‐experimental designs were included. We performed an overall random‐effect meta‐analysis and subgroup analyses.

**Results:**

We found a significant moderate overall effect of non‐pharmacological interventions on challenging behaviours (*d* = 0.573, 95% CI [0.352–0.795]), and this effect appears to be longlasting. Interventions combining mindfulness and behavioural techniques showed to be more effective than other interventions. However, this result should be interpreted with care due to possible overestimation of the subgroup analysis. No differences in effectiveness were found across assessment times, delivery modes or study designs.

**Conclusions:**

Non‐pharmacological interventions appear to be moderately effective on the short and long term in reducing challenging behaviours in adults with intellectual disabilities.

## 1 Introduction

Non‐pharmacological interventions for challenging behaviours of adults with intellectual disabilities are being recommended as first line treatments by several leading clinical guidelines (Banks & Bush, 2016; National Institute for Health and Care Excellence, 2017; Sullivan *et al.,* 2018). Moreover, health care professionals prefer non‐pharmacological interventions to pharmacological treatments for the management of challenging behaviours (Unwin & Deb, 2008). However, the evidence on the effectiveness of non‐pharmacological interventions for challenging behaviours of adults with intellectual disabilities remains unclear. In the past decades, much of the intervention research focused on children and adolescents rather than on adults (Brosnan & Healy, 2011; Heyvaert, Meas, & Onghena, 2010; McIntyre, Blacher, & Baker, 2002), and concerned studies that lacked follow‐up measures (Brosnan & Healy, 2011; Chan *et al.,* 2010), with small sample sizes (Didden, Korzillus, van Oorsouw, & Sturmey, 2006; Hassiotis & Hall, 2008; Heyvaert, Maes, van den Noortgate, Kuppens, & Ongehena, 2012) and uncontrolled designs (Allen & Tynan, 2000). Only recently, studies with larger adult sample sizes and (randomised) control groups have been published (Hassiotis *et al.,* 2018; MacDonald, McGill, & Murphey, 2018; McGill *et al.,* 2018; Singh *et al.,* 2018). These studies have not yet been included in the most recent meta‐analysis (Knotter *et al.,* 2018), which found that staff training does not reduce challenging behaviours of individuals with intellectual disabilities. Combining early and more recent findings is warranted, in order to gain reliable and up to date insight into the effectiveness of non‐pharmacological interventions.

Approximately 10–20% of adults with intellectual disabilities show challenging behaviours (Emerson *et al*. 2001; Bowring, Totsika, Hastings, Toogood, & Griffith, 2017), including aggression, disruptive and socially inappropriate behaviours, self‐injury and withdrawal behaviours (Hartley & MacLean, 2007; Lundqvist, 2013). They can be long‐lasting and harmful for the quality of life of the individual concerned (Cooper *et al.,* 2009; Heyvaert *et al.,* 2010). Individuals with intellectual disabilities and challenging behaviours are at higher risk of abuse, neglect, deprivation, institutionalisation, and physical and chemical restraints, compared to individuals with intellectual disabilities without challenging behaviours (Sturmey, 1999; Emerson *et al*., 2001; Robertson *et al.,* 2005; Holden & Gitlesen, 2004). Besides, challenging behaviours may negatively affect the immediate environment of the individual concerned. Caregivers may be subjected to verbal and physical abuse, or to witnessing self‐injurious behaviours (Lambrechts & Maes, 2009). These experiences may cause anxiety, anger, fear and emotional exhaustion (Allen & Tynan, 2000; Smyth, Healy, & Lydon, 2015; Strand, Benzei, & Saveman, 2004). Additionally, staff working with individuals with intellectual disabilities and challenging behaviours report to feel impaired in providing sufficient care (Hartley & MacLean, 2007). Therefore, it is of the utmost importance to treat these behaviours.

The evidence for the effectiveness of non‐pharmacological interventions to reduce challenging behaviours in adults with intellectual disabilties is ambiguous. Whereas some previous reviews and meta‐analyses found that non‐pharmacological interventions are indeed effective in reducing challenging behaviours (Brosnan & Healy, 2011; Didden *et al.,* 2006; Harvey, Boer, Meyer, & Evans, 2009; Heyvaert *et al.,* 2010; Heyvaert *et al.,* 2012; Shogren, Faggella‐Luby, Jik, & Wehmeyer, 2004), others did not (Gustafsson *et al*., 2009; Hassiotis & Hall, 2008; Chan *et al.,* 2010; Cox, Dube, & Temple, 2015; Knotter *et al.,* 2018). These contradictory findings may be due to the scarcity of high quality studies included in previous reviews and meta‐analyses. Another explanation might be the heterogeneity in non‐pharmacological interventions, as these include various treatments of different theoretical backgrounds. Examples include treatments directed at the individual such as multisensory therapy, mindfulness or cognitive behavioural therapy (CBT; Hassiotis & Hall, 2008; Lotan & Gold, 2009; Chan *et al.,* 2010; Hwang & Kearney, 2013; Nicoll, Beail, & Saxon, 2013), and interventions directed at the environment, such as staff training, applied behaviour analysis (ABA), positive behaviour support or specialised teams (Hassiotis *et al.,* 2009; Knotter *et al.,* 2018; LaVigna & Willis, 2012; MacDonald & McGill, 2013). Moreover, some non‐pharmacological interventions are adapted to the specific individual and his or her context, usually by means of a functional analysis of the behaviour of the individual (e.g. ABA or positive behaviour support), while others are more generic programs (e.g. multisensory therapy). Recent studies found positive effects of environmentally mediated positive behaviour support with or without mindfulness components (MacDonald *et al.,* 2018; McGill *et al.,* 2018; Singh *et al.,* 2018). These studies were published after the most recent meta‐analysis, which found that staff training has no effect on challenging behaviours of adults with challenging behaviours (Knotter *et al.,* 2018).

The current study was primarily aimed at updating the existing evidence on the effectiveness of non‐pharmacological interventions to treat challenging behaviours in adults with intellectual disabilities. Secondary aims were to investigate long term treatment effects, and to examine whether intervention type (i.e. interventions of different theoretical backgrounds) and delivery mode (i.e. individual interventions or environment mediated interventions) were associated with differences in treatment effects. Furthermore, we aimed to investigate whether study design (i.e. randomised versus non‐randomised) was related to differences in outcome.

## 2 Method

### 2.1 Registration and literature search

The current meta‐analysis was registered at PROSPERO (registration number: CRD42016051263; https://www.crd.york.ac.uk/PROSPERO/display_record.php?RecordID=51263). We included studies that 1) reported on the evaluation of one or more non‐pharmacological intervention(s), primarily aimed at reducing or eliminating challenging behaviours of adults with intellectual disabilities (regardless of other diagnoses); 2) included a sample with at least 75% of participants of 18 years or older; 3) used an experimental design (randomised controlled trial; RCT) or quasi‐experimental design (pretest‐posttest or controlled study) with at least 15 participants; 4) were English‐written; 5) were published in an academic, peer‐reviewed journal; 6) contained sufficient data to perform meta‐analyses (i.e. pre and posttest means, standard deviations, sample sizes, and odds ratios and/or correlations).

In order to be able to investigate a rather homogeneous sample of adults with intellectual disabilities and challenging behaviours, we excluded studies in forensic settings or with forensic participants. Delinquent adults with intellectual disabilities differ in aggression levels compared to non‐delinquent adults with intellectual disabilities (Nicoll & Beail, 2013). By excluding the forensic population, our results would be more specifically applicable to the general care for adults with intellectual disabilities and challenging behaviours.

We used the EBSCOHOST databases Medline, Eric, PsychINFO and Cinahl and searched echt electronic database separately, after which duplicates were removed. Furthermore, reference lists of relevant systematic reviews and meta‐analyses were hand‐searched to check for possible missing articles. We completed the search on November 14^th^ 2019. Table 1 displays the search terms used for the databases. Only two limits were applied: publication type (academic journals only) and the publication language (English).

**Table 1:** Overview search terms

Population	Dependent variable	Actions to alter behaviour
cognitive impair*	behavio* AND problem*	therap*
mental* AND retard*	tantrums	treat*
intellectual* AND disab*	aggressi*	interven*
learning AND disab*	self‐inju*	behavio* AND modification
developmental* AND disab*	self‐inflicted AND wounds	training
adult	Self‐mutilation	applied behavio* analysis
elderly	stereotyp*	positive AND behavio* AND support
individual	challenging AND behavio*	
	problem AND behavio*	
	aggressive AND behavio*	
	aberrant AND behavio*	
	provocative AND behavio*	
	stereotyped AND behavio*	
	repetitive AND behavio*	
	disruptive AND behavio*	
	destructive AND behavio*	
	maladaptive AND behavio*	

The first author (EB, PhD student) screened all search results on their eligibility in a three‐step process: screening based on the title, based on the abstract, and based on the full text paper. The excluded articles were checked by the last author (AdB, senior researcher) and disagreement was resolved through consensus. If agreement could not be achieved, the second author (BJvdH, professor) was consulted. Data extraction was done by the first author. In case data were insufficiently described in the paper, authors were contacted by e‐mail or through Researchgate (https://www.researchgate.net/). The following study characteristics were recorded from the included studies: 1) participant characteristics (level of intellectual disability and age range); 2) intervention characteristics (intervention type and content, directed at individual or staff, and number of sessions); 3) number of participants, comparison groups, and design; and 4) outcome measures.

All included studies were assessed by the first author (EB) on potential sources of bias: random sequence, allocation concealment, blinding of outcome assessment, incomplete outcome data, selective reporting, group similarity at baseline, and personal or financial gain (Higgins & Green, 2008). Additionally, the next step was the comparison between the effect sizes of studies with a low risk of bias and the effect sizes of studies with a high risk of bias through subgroup analysis.

### 2.2 Data analyses

Because we assumed that the true effect would vary between studies, we used the random effect model to calculate the summary effect (Borenstein, Hedges, Higgins, & Rothstein, 2009) using the software Comprehensive Meta‐Analysis (CMA) Version 2.0 (Borenstein *et al.,* 2009). The summary effect was expressed as the overall standard difference in means (Cohen's *d*). A Cohen's *d* of 0.2 was considered small, 0.5 moderate, and 0.8 large (Cohen, 1988). We generated a forest plot of the overall random‐effect of interventions and measured heterogeneity with *I* ^*2*^. The percentage of *I* ^*2*^ describes the variability that is due to heterogeneity rather than sampling error (Borenstein *et al.,* 2009). Values around 25% are considered low, 50% is considered moderate and 75% is considered high (Higgins, Thompson, Deeks, & Altman, 2003). To perform the random‐effects meta‐analysis, we held to the following assumptions: 1) if test–retest correlation of instruments was not specified in the paper, we used a correlation of *r* = 0.5; 2) if a study contained multiple parameters measuring different challenging behaviours, we used a summarised measure for the calculation of an overall challenging behaviours measure (‘Use the mean of the selected outcomes’ option of Comprehensive Meta‐Analysis software); 3) if a studied intervention resulted in significant improvement of behaviours, the direction of the effect was stated positive; 4) data were standardised by post score standard deviations (SD); and 5) in case of multiple follow‐up time points, these were computed together as a single measure. We conducted a sensitivity analysis using ‘one study removed analyses’ (Borenstein *et al.,* 2009), to investigate the robustness of our results.

We performed four subgroup analyses to examine differences in treatment effects across assessment times, intervention types, delivery modes and study designs. For the first analysis, we compared post intervention assessments with follow‐up assessments, to examine long‐term effectiveness. Second, we categorised all included interventions into five intervention types, based on their theoretical background: 1) ABA or behavioural interventions, 2) cognitive behavioural therapy (CBT), 3) interventions combining mindfulness and behavioural techniques, 4) multisensory therapy, and 5) specialised teams using personalised treatment plans (i.e. Invididualized Habituation Plan (IHP)). All categories (i.e. intervention type) were compared on effectiveness. In the third and fourth subgroup analyses we compared interventions directly aimed at the individual with environment mediated interventions, and RCTs with non‐RCTs, respectively.

Finally, to examine possible publication bias we generated a funnel plot (Duval & Tweedie, 2000) and used the Duval and Tweedie's trim and fill option to detect missing studies in the funnel plot.

## 3 Results

### 3.1 Study characteristics

**Figure 1:**
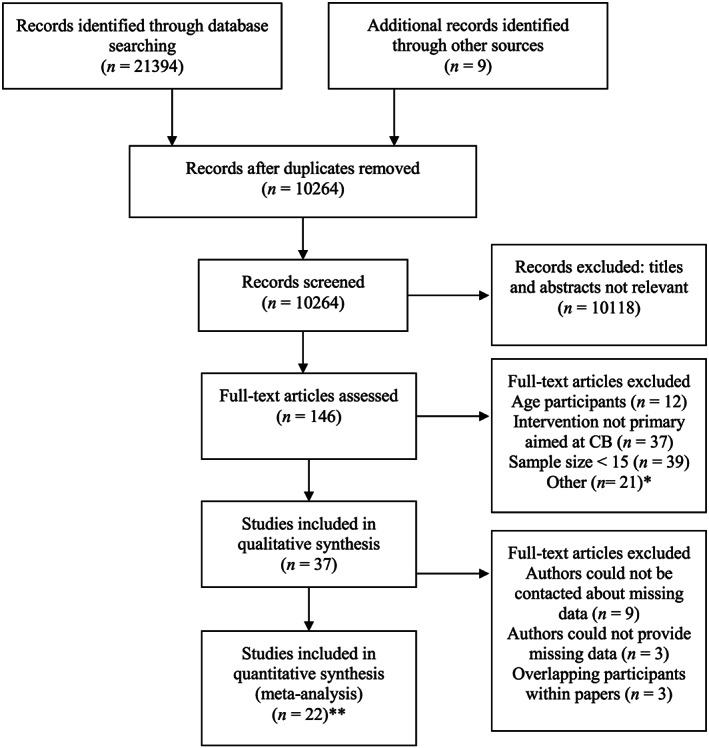
PRISMA flowchart of the screening process.

The combination of the electronic search and reference tracking resulted in 10264 titles. After the three‐step screening procedure 22 studies were included for this meta‐analysis. The complete selection procedure is illustrated in Figure 1.

The PRISMA flowchart shows a distinction between ‘studies included in qualitative synthesis’ and ‘studies included in quantitative analysis’. Studies included in our qualitative synthesis met our inclusion criteria, but did not report on data necessary to perform a meta‐analysis. To gain missing data, authors were contacted by e‐mail. Unfortunately, this was unsuccessful: authors of five papers could not be reached due to outdated contact information (Azrin & Wesolowski, 1974; Bhaumik *et al.,* 2009; Parsons & Reid, 1993; Tyson & Spooner, 1991; Williams, Kirkpatrick‐Sanchez, Enzinna, Dunn, & Borden‐Karasck, 2009); authors of four papers did not respond to our requests (Bodfish & Konarski, 1992; Comaty, Stasio, & Advokat, 2001; Lowe, Felce, & Blackman, 1996; Xenitidis, Henry, Russell, Ward, & Murphy, 1999); and authors of three papers whom we contacted could not provide the necessary data (Benson, Johnson, & Miranti, 1986; Hassiotis *et al.,* 2009; Singh *et al.,* 2006). Three papers were excluded from the quantitative analysis due to overlapping participants between studies (Rose, 2010; Rose, 2013; Rose, O'Brien, & Rose, 2009).

Together, the 22 included studies contained 1676 participants. An overview of all characteristics (i.e. participant characteristics, design, outcome parameters) and intervention characteristics (i.e. content, directed at individual or staff, number of sessions, intervention type) is presented in Table 2.

**Table 2:** Study and intervention characteristics of studies included in the meta‐analysis

**Study**		***Participant characteristics***			***Intervention characteristics***		***Study characteristics***		
	**Level ID**	**Age range**	**Intervention**	**Content**	**Directed at individual or environment**	**Intervention type**	***n***	**Design**	**Outcome measure**
Brown, Brown, & Dibiasio, 2013	IQ range [40–95]	19–63 years	Dialectical Behaviour therapy (DBT)	Incorporated core strategies of Applied Behaviour Analysis (ABA), Cognitive‐behavioural Therapy (CBT) and mindfulness.	Individual	Intervention combining mindfulness, behavioural and cognitive behavioural therapy techniques	40	Pretest‐posttest design	Red Flag behaviours & Dangerous Situations
Chan, Fung, Tong, & Thompson, 2005	Mild, moderate and severe	11–71+ years (92% of participants 118 or older)	Multisensory therapy	Induces leisure, enjoyment and relaxation through enhancing sensations and emotions.	Individual	Multisensory therapy	89	Randomised controlled trial	Checklist of Challenging Behaviour
Evans & Berryman, 1998	Severe to moderate	18–69 years	Non‐aversive behaviour management	Focused on teaching alternative responses to enhance adaptive lifestyles in the naturalistic setting.	Environment	Behavioural intervention	18	Randomised controlled trial	Clinician's rating of treatment effectiveness
Hagiliassis, Gulbenkoglu, Di Marco, Young, & Hudson, 2005	Borderline, mild, moderate and severe	26–74 years	Group anger management	Within the framework of the cognitive‐behavioural framework of anger, relevant content, activities and techniques are adopted.	Individual	CBT	29	Randomised controlled trial	Novaco Anger Scale (NAS)
Hassiotis *et al.,* 2018	Mild, moderate and severe	25–51 years	Positive behaviour support	A multicomponent approach for helping the direct environment to better understand an individual's challenging behaviour, and to apply a personalised approach.	Environment	Behavioural intervention	245	Multicenter, cluster randomised controlled trial	Aberrant Behaviour Checklist (ABC)
Lundqvist, Andersson, & Viding, 2009	Mild, moderate and severe	22–57 years	Vibroacoustic music	The vibroacoustic music enhances feelings of relaxation.	Individual	Multisensory therapy	20	Randomised controlled trial	The Behaviour Problems Inventory (BPI)
MacDonald *et al.,* 2018	Unknown	18–63 years	Positive behaviour support for managers	Teaches managers to develop function based positive behaviour plans and then lead their staff team in implementing these plans.	Environment	Behavioural intervention	72	Non‐randomised control group	Aberrant Behaviour Checklist (ABC)
Martin, Gaffan, & Williams, 1998	Severe and profound	22–61 years	Snoezelen	Multiple sensory stimulations which result in relaxing and stimulating sensations.	Individual	Multisensory therapy	27	Double crossover design	Problem Behaviour Inventory (PBI)
McClean *et al.,* 2005	Mild, moderate, profound, and severe	105 adults and 33 children aged 19 or under (76% of participants 18 and older)	Person Focused Training	A staff training focused on designing and implementing a multi‐element behaviour support plan.	Environment	Behavioural intervention	138	Pretest‐posttest with follow‐up	Target behaviour selected as part of the Person Focused Training
McClean & Grey, 2012	Mild, moderate, severe, and profound	7–54 years (80% of participants 18 and older)	Positive Behaviour Support	Aimed at teaching staff members to conduct background assessment, functional assessment, intervention design, and implementation.	Environment	Behavioural intervention	61	Pretest‐posttest with follow‐up	The Challenging Behaviour Rating Scales
McGill *et al.,* 2018	Unknown	19–84 years	Setting‐wide Positive Behaviour Support	Focused on the context in which challenging behaviours occur. Furthermore, staff learned how to develop function based positive behaviour plans.	Environment	Behavioural intervention	81	Cluster randomised controlled trial	Aberrant Behaviour Checklist (ABC)
Roeden, Maaskant, & Curfs, 2014	IQ range [50–70]	18–60 years	Solution‐ Focused Brief Therapy	A short‐term, goal‐focused and client‐directed therapeutic approach that aimed to help individuals with intellectual disabilities to focus on solutions rather than on problems.	Individual	Behavioural intervention	38	Controlled pretest‐posttest design	Reiss Screen for Maladaptive Behaviour (RSMB)
Rose, West, & Clifford, 2000	Participants had to have a degree of receptive language such that they could understand simple directions	20–62 years	Group‐based anger management	Psycho‐educational approach that provided individuals with ID with instructions on how to work on emotional recognition, the causes and manifestation of anger, coping and preventative strategies, and problem solving.	Individual	CBT	41	Controlled pretest‐posttest design	Anger Inventory
Rose, Dodd, & Rose, 2008	Participants had a degree of receptive language such that they could understand simple directions	Mean age intervention group: 37.05; mean age waiting list: 37.14	Cognitive Behavioural Intervention for anger	Provided individuals with ID with instructions on how to work on emotional recognition, the causes and manifestation of anger, coping and preventative strategies, and problem solving.	Individual	CBT	41	Controlled pretest‐posttest design	Anger inventory
Singh *et al.,* 2004	Severe & profound	22–57 years	Snoezelen	Multiple sensory stimulations which result in relaxing and stimulating sensations.	Individual	Multisensory therapy	45	Repeated measures counter‐balanced design	Aggressive acts & self‐injurious behaviour
Singh *et al.,* 2013	Borderline, and mild	17–31 years (91% of participants 18 or older)	Mindfulness ‐ based treatment of aggression	Aimed to teach individuals with ID to recognise precursors of aggressive behaviour, to disengage their attention from the precursors, and to redeploy their attention to a neutral point on the body, the soles of their feet.	Individual	Intervention combining mindfulness and behavioural techniques	34	Randomised controlled trial	Physical aggression & verbal aggression
Singh *et al*. 2016a	Mild	18–37 years	Mindfulness‐Based Positive Behaviour Supports	Aimed to teach new skills to individuals with ID and to modify the environment where the challenging behaviour occurs, combined with a stress reduction program for staff members.	Environment	Intervention combining mindfulness and behavioural techniques	18	Pretest‐posttest	Staff injury and Peer injury
Singh *et al*. 2016b	Severe & profound	24–57 years	Caregiver Training in Mindfulness‐based Positive Behaviour Support	Combined components of standard Positive Behaviour Support with mindfulness to regulate emotions during periods of acute stress.	Environment	Intervention combining mindfulness and behavioural techniques	48	Randomised controlled trial	Aggressive events
Singh *et al.,* 2018	Mild & Moderate	24–63	Caregiver Training in Mindfulness‐Based Positive Behaviour Support	Combined Standard Positive Behaviour Support with Mindfulness components to teach caregivers ways of managing their psychological distress.	Environment	Intervention combining mindfulness and behavioural techniques	80	Randomised controlled trial	Aggressive events, Staff injury, Peer injury
Stancliffe, Hayden, & Lakin, 1999	Mild, moderate, severe, and profound	Unknown	Individualised Habilitation Plan (IHP)	Aimed to improve personalised plans, expressing behavioural problems, described interventions used, completion date and specification of methods.	Environment	Individualised Habilitation Plan	130	Pretest‐posttest design	General Maladaptive Index
Tyrer *et al.,* 2017	Mild, moderate, and severe	17–70 years (99.5% of participants 18 or older)	Nidotherapy	Aimed at performing antecedent interventions.	Environment	Behavioural intervention	200	Cluster randomised controlled trial	Problem Behaviour Checklist (PBCL)
Willner *et al.,* 2013	IQ range [53–64]	27–48 years	Group‐based cognitive‐behavioural anger management	Aimed to teach individuals with ID to be aware of situations that evoke anger, to be aware of becoming angry, and to develop skills to control and manage anger.	Individual	CBT	181	Cluster randomised trial	Aberrant Behaviour Checklist – Irritability (ABC‐I) & Hyperactivity (ABC‐H)*

Note: We solely used the keyworkers data of the Aberrant Behavior Checklist. Namely, the majority of included papers used keyworkers /staff members as informants. Additionally, some data of the home carers were missing

We solely used the keyworkers data of the Aberrant Behavior Checklist. Namely, the majority of included papers used keyworkers /staff members as informants. Additionally, some data of the home carers were missing.

**Figure 2:**
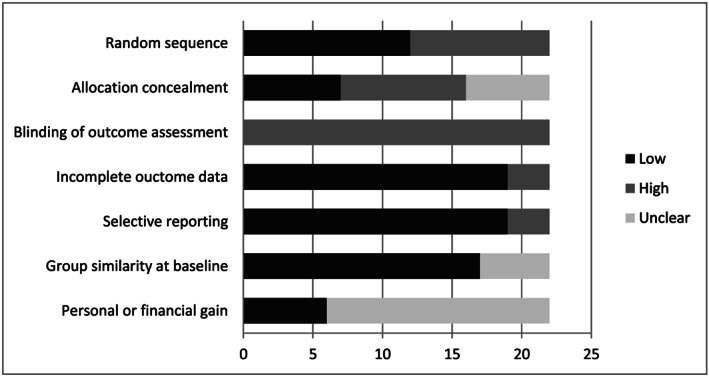
Overview of the risk of different sources of bias of the included studies (number of studies presented on the x‐axis).

Figure 2 shows an overview of the risk of different sources of bias of the included studies. Information on ‘personal or financial gain’ was too often missing to draw conclusions on. Additionally, due to limited variation in sources of bias between studies and frequent ‘unclear’ scores we had to refrain from comparing the effect sizes of studies with low risks of bias to the effect sizes of studies with high risk of bias.

### 3.2 Meta‐analysis

**Figure 3:**
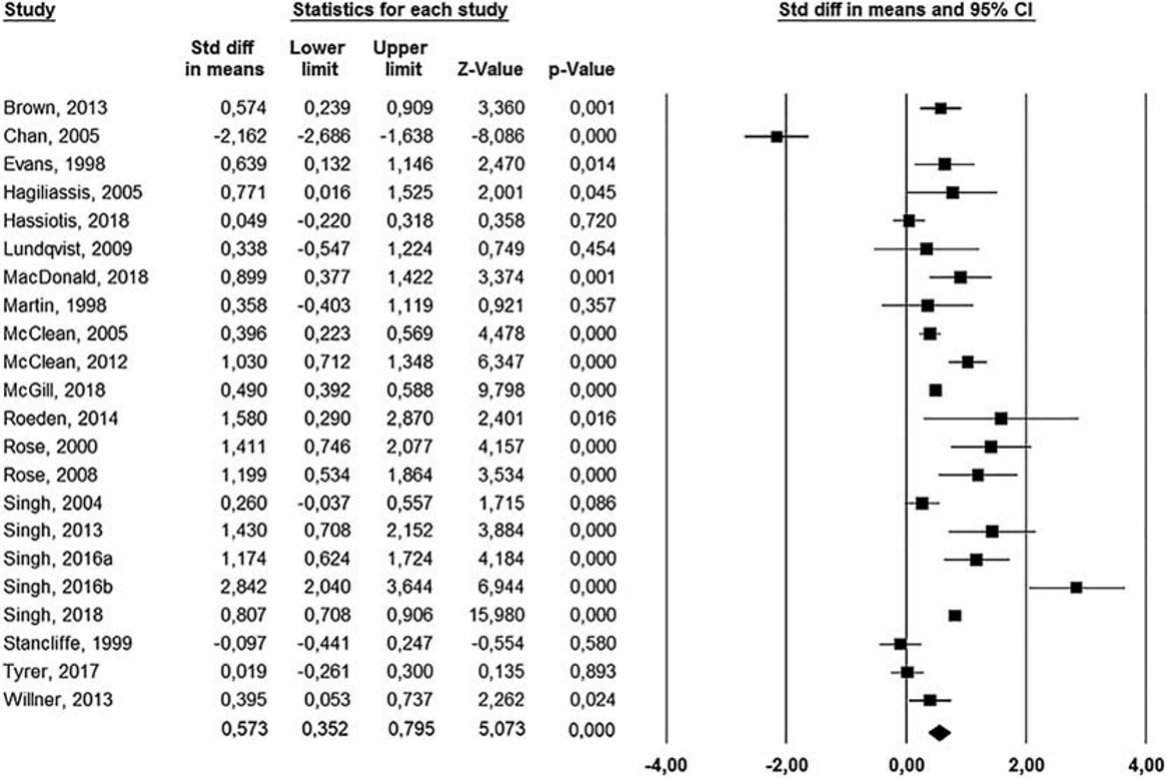
Forest plot of overall random‐effect of non‐pharmacological interventions.

The random‐effects model showed an overall treatment effect with a moderate effect size (*d* = .573, *P <* .001, CI [0.352, 0.795]). The individual and combined effect sizes, lower limits, upper limits, z‐values and p‐values are presented in Figure 3. Heterogeneity was high (*I* ^*2*^ = 91.40%). The sensitivity analyses showed that the effect sizes varied between 0.491 and 0.666. These values fall within the range of the confidence interval of the overall effect size, indicating that our results were robust.

### 3.3 Subgroup analyses

We found no significant differences between post‐intervention assessments versus follow‐up assessments (*Q* = 0.198, d.f. = 1, *P* = 0.656). There was however a significantly higher effect of interventions combining mindfulness and behavioural techniques than of all other intervention types (*Q* = 9.176, d.f. = 1, *P* = 0.002). There were no significant differences between behavioural interventions versus all other intervention types (*Q* = 0.871, d.f. = 1, *P* = 0.351), or CBT versus all other intervention typers (*Q =* 1.540*,* d.f. = 1, *P* = 0.215). Furthermore, we found no significant differences between individual interventions ersus environment mediated interventions (*Q* = 0.132, d.f. = 1, *P* = 0.717), and RCTs controlled designs versus non‐RCTs designs (*Q* = 2.136, d.f. = 1, *P* = 0.144).

### 3.4 Publication bias

**Figure 4:**
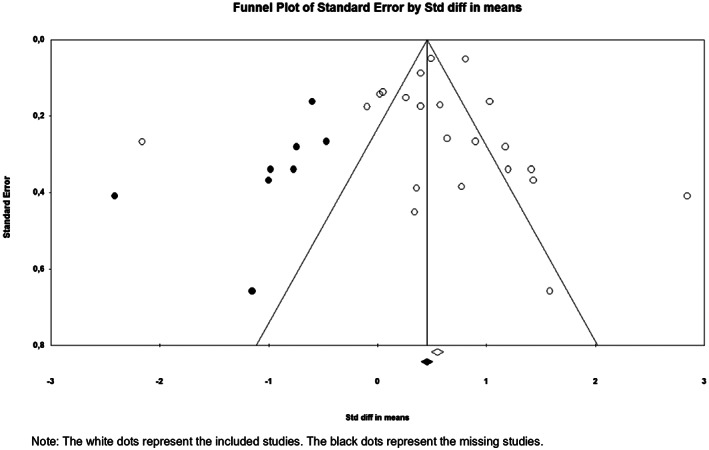
Non‐pharmacological interventions and challenging behaviours – funnel plot.

The funnel plot (Figure 4) shows clear asymmetry, with a predominance of papers on the right range of the plot (displayed as white dots in Figure 4), suggesting publication bias. The unequal distribution of effect sizes of our included studies was confirmed by the Duval and Tweedie's trim and fill analysis. The eight black dots on the left side of the plot represent expected studies with negative effect sizes that were not included in the meta‐analysis. This finding suggests that there may have been studies that have not been published.

## 4 Discussion

This meta‐analysis provides insight in the effectiveness of non‐pharmacological interventions to treat challenging behaviours in adults with intellectual disabilities. We found a moderate overall effect of non‐pharmacological interventions, consistent with some previous meta‐analyses (Harvey *et al.,* 2009; Heyvaert *et al.,* 2010; Shogren *et al.,* 2004). However, some other reviews and meta‐analyses did not find evidence for the effectiveness of non‐pharmacological interventions (Gustafsson *et al*. 2009; Hassiotis & Hall, 2008; Chan *et al.,* 2010; Nicoll *et al.,* 2013). This difference in findings may be due to the different aims of previous reviews and meta‐analyses. For example, the meta‐analysis of Nicoll *et al*. (2013) was specifically aimed at cognitive behavioural treatment for anger in adults with challenging behaviours and intellectual disabilities, while the meta‐analysis of Heyvaert *et al*. (2010) more broadly examined pharmacological, psychotherapeutic, and contextual interventions for treating challenging behaviours in individuals with intellectual disabilities.

Our results indicate that effect sizes of non‐pharmacological interventions are also moderate effective on the long‐term (follow‐up measures ranged from 3 to 18 months), suggesting that treatment effects of non‐pharmacological interventions sustain after the intervention has ended. However, we must be cautious with the interpretation and implications of this finding, as the measures of post intervention assessments and follow‐up assessments are not independent from eachother. To our knowledge, there have been no earlier studies that have compared post‐intervention effects with follow‐up effects. Currently, in clinical practice, pharmacological treatments, instead of non‐pharmacolocial interventions, are often the first treatment of choice (Holden & Gitlesen, 2004). This may be due to the immediate effects of medication, in contrast to the gradual effects of non‐pharmacological interventions (Beadle‐Brown, Mansell, Whelton, Hutchinson, & Skidmore, 2006). Moreover, non‐pharmacological interventions often require a substantial time investment of health care professionals (Matson & Wilkins, 2008). However, the use of medication is controversial due to negative side effects (Matson & Mahan, 2010; Sheehan *et al.,* 2017) and questionable effectiveness (Scheifes *et al.,* 2016; Shankar, Wilcock, Oak, McGowan, & Sheehan, 2019; Sheehan *et al.,* 2015). The possible long‐term positive outcomes we found of non‐pharmacological interventions might motivate clinicians to invest in non‐pharmacological interventions more often, rather than medication.

Interventions combining mindfulness with behavioural techniques showed to be more effective than behavioural interventions without mindfulness components, CBT, multisensory therapy, and individualised habituation plans. No previous studies have demonstrated the superiority of this type of interventions (Heyvaert *et al.,* 2010; Hwang & Kearney, 2013). However, this finding should be interpreted with care. Subgroup analyses may be misleading, due to missing randomised comparisons, which makes the results more susceptible to false positive tests results (Higgins & Green 2008). Moreover, all included studies reporting on interventions combining mindfulness and behavioural techniques came from the same research group. RCTs from other research groups, with head to head comparisons, are necessary to draw more robust conclusions on the effects of those interventions on challenging behaviours of adults with intellectual disabilities.

We found no differences in effect between individual directed interventions and environment mediated interventions. Earlier reviews and meta‐analyses demonstrated that interventions that were aimed at altering the environment, or at training staff were effective (Brosnan & Healy, 2011; Heyvaert *et al.,* 2010; Heyvaert *et al.,* 2012), while other reviews and meta‐analyses did not (Cox *et al.,* 2015; Knotter *et al.,* 2018; van Oorsouw, Embregts, Bosman, & Jahoda, 2009). Our results indicate that there are no differences in effect sizes between interventions aimed at the environment versus at the individual. However, there are clear differences in applicability of individual directed interventions versus environment mediated interventions. For instance, to conduct CBT, the individual needs the verbal skills to express feelings and thoughts (Sturmey, 2004) which is only the case in higher functioning individuals with intellectual disabilities. In contrast, environment mediated interventions, such as staff training, are more broadly applicable to individuals with different levels of intellectual disabilities. Such interventions provide staff with tools that they can use more consistently, and apply in new situations, possibly indicating a more sustainable effect. However, implementing such environment mediated interventions is known to be a struggle (Bosco *et al.,* 2019). Insufficient training and supervision, high turnover rates, time constraints and low support from management have shown to be pitfalls in implementing environment mediated interventions (Bosco *et al.,* 2019; Campbell, 2010). As a consequence, the risk of ineffective treatment increases (Feldman, Atkinson, Foti‐Gervais, & Condillac, 2004).

In line with the meta‐analysis of Heyvaert *et al*. (2010), we did not find differences in effect sizes between RCTs and non‐RCTs, indicating no evidence for overestimation of treatment effect of non‐RCTs. It is interesting however, that the number of RCTs in the field on non‐pharmacological intervention studies appears to be rising. Previous reviews and meta‐analyses reported a scarcity of methodologically sound clinical trials in the field on non‐pharmacological intervention studies for adults with intellectual disabilities and challenging behaviours (Gustafsson *et al*. 2009; Hassiotis & Hall, 2008; Nicoll *et al.,* 2013). In our meta‐analysis, the balance between RCTs (*n* = 11) and non‐RCTs (n = 11) was more even than in earlier ones (Heyvaert *et al.,* 2010; including 5 RCTs against 10 non‐RCTs; Nicoll *et al.,* 2013; including 2 RCTs against 10 non‐RCTs). The increasing number of RCTs is promising, especially because conducting clinical trials in the field of non‐pharmacological intervention studies for adults with intellectual disabilities and challenging behaviours is known to be challenging (Cleaver *et al*. 2010; Robotham *et al*. 2011; Nicholson, Colyer, & Cooper, 2013). Many clincial trials experienced recruitment problems, high drop out rates and high staff turnover (Bhaumik, Gangadharan, Hiremath, & Russel, 2011; Hassiotis *et al.,* 2018). Only recently was the first paper on process evaluation of a non‐pharmacological intervention study (e.g. positive behaviour support) published (Bosco *et al.,* 2019), showing that participants found it difficult to combine trial required assessments with routine clinical care. More of these process evaluations are warranted, as they increase insight in the specific barriers of conducting clinical trials in the field of adults with intellectual disabilities. Findings may help prevent such problems for future studies or to apply more flexible trial designs.

Previous studies indicated that interventions applying functional analysis were more effective than interventions which did not incorporate this (Didden *et al.,* 2006; Harvey *et al.,* 2009; Brosnan & Healy, 2011; Heyvaert *et al.,* 2012; Lydon *et al*. 2013; Lloyd & Kennedy 2014). Moreover, the use of functional analysis is recommended by clinical guidelines (Banks & Bush, 2016; National Institute for Health and Care Excellence, 2019). Unfortunately, in our meta‐analysis we were unable to analyse whether intervention effects differed in this respect, as some of the included papers were ambiguous about the incorporation of assessment of function in the intervention.

Important to note is the high heterogeneity we found, which indicated that most of the observed variance was real. However, our sensitivity analysis showed that the effect sizes all stayed within the range of the confidence interval of the overall moderate effect size, indicating that our results were robust. Moreover, we anticipated that the true effect sizes would vary. Hence we conducted a random effect model, which is more conservative than the fixed effect model (Fletcher, 2007). Furthermore, the overall effect of our study is in line with previous, broad aimed meta‐analyses which compared wide ranges of interventions (Didden *et al.,* 2006; Heyvaert *et al.,* 2010; Heyvaert *et al.,* 2012). Therefore, we believe that our results are a valuable addition to the body of evidence on the effectiveness of non‐pharmacological interventions. Another important finding of our study was publication bias we found. Our results showed that especially large scale trials reporting no or negative effects were missing. Some previous meta‐analyses also detected publication bias (Denis, van den Noortgate, & Maes, 2011; Heyvaert *et al.,* 2012), while others did not (Hart & Banda, 2010; Heyvaert *et al.,* 2010; Knotter *et al.,* 2018). Since we only included English‐written papers, we expected a certain level of publication bias. In the future, consequent registration of trials is important to bring about more transparency on studies and reduce publication bias.

The strength of our study was the number of studies that conducted large scale RCTs. However, our findings should also be interpreted in light of its limitations. A first limitation was the exclusion of eligible studies due to missing data or missing papers. Despite our efforts to collect all necessary data and papers, we could not get in touch with some authors, or the authors could not provide us with the necessary data, and we therefore had to exclude their studies (*n* = 12). The exclusion of approximately a third of the eligible papers increased the risk of bias and may have affected our results. Second, we did not include single‐case studies in our meta‐analysis. This resulted in a loss of papers, especially from earlier research on interventions for challenging behaviours within the population of individuals with intellectual disabilities. However, we chose to include only studies with experimental or quasi‐experimental designs, in order to update and build upon previous meta‐analyses of studies using these kind of designs (Heyvaert *et al.,* 2010; Knotter *et al.,* 2018). This approach also had the advantage of being able to analyse a methodologically more homogeneous group of studies, compared to meta‐analyses including small‐*n* designs as well (e.g., Nicoll *et al.,* 2013). Third, ‘non‐pharmacological interventions’ could have been a too broad range of different interventions to cluster together for an overall effect, and indeed our results showed high heterogeneity. However, as previous stated, past meta‐analyses have also included a broad range of interventions (e.g. Didden *et al.,* 2006; Heyvaert *et al.,* 2010; Heyvaert *et al.,* 2012), which enhances the comparablility of our study with these studies. Fourth, the population that we examined (i.e. adults with intellectual disabilities) was quite heterogeneous. We included studies on individuals with all levels of intellectual disability (profound to borderline) and a broad age range. Unfortunately, we were unable to collect individual participant data (i.e. level of intellectual disability and age) of the included studies, therefore we could not analyse the effect of these characteristics on intervention effects and heterogeniety. Future effectiveness studies should focus on how and which participant characteristics affect treatment success (i.e. level of intellectual disability, age). Finally, we only examined the reduction of challenging behaviours as a measure of treatment success. While challenging behaviours have far reaching negative consequences, for the individuals with intellectual disabilities as well as their environment, future studies should take quality of life of the individual with intellectual disabilities, or emotional wellbeing of staff into account as other relevant parameters in the evaluation of treatment effectiveness.

In conclusion, we found a moderate effect of non‐pharmacological interventions in reducing challenging behaviours in adults with intellectual disabilities, and this effect appears to be longlasting. To assess the superiority of different types of interventions, more research is needed. Fortunately, there is a positive development in the scientific field with the growing numbers of large scale, RCTs that are being conducted. For future research, trial registration and conducting more large scale studies with high quality designs is necessary. Furthermore, future studies should examine the effect of participant characteristics on treatment success, such as level of intellectual disability and age, and take other outcome measures into account, such as quality of life or staff wellbeing. These steps will add to a more comprehensive perspective on the effect of non‐pharmacological interventions.

## Conflict of interest

No conflict of interests have been declared.

## Funding

This study was funded by the Dutch Research Council (NWO; grant number 432‐13‐809).
